# Precipitating and relieving factors of migraine versus tension type headache

**DOI:** 10.1186/1471-2377-12-82

**Published:** 2012-08-25

**Authors:** Badrul Haque, Kazi Mohibur Rahman, Azharul Hoque, ATM Hasibul Hasan, Rajib Nayan Chowdhury, Sharif Uddin Khan, Mondal Badrul Alam, Mansur Habib, Quazi Deen Mohammad

**Affiliations:** 1 Department of Neurology, Dhaka Medical College Hospital, Dhaka, Bangladesh; 2 Department of Medicine, Dhaka Medical College Hospital, Dhaka, Bangladesh

**Keywords:** Headache, Tension type headache (TTH), Migraine

## Abstract

**Background:**

To determine the differences of precipitating and relieving factors between migraine and tension type headache.

**Methods:**

This is a cross sectional study. We retrospectively reviewed the records of 250 migraine patients and 250 patients diagnosed as tension type headache from the specialized headache clinic in Dept. of Neurology, Dhaka Medical College Hospital. Data were collected through a predesigned questionnaire containing information on age, sex, social status and a predetermined list of precipitating and relieving factors.

**Results:**

In this study, the female patients predominated (67%). Most of the patients were within 21–30 years age group (58.6%). About 58% of them belonged to middle class families. The common precipitating factors like stress, anxiety, activity, journey, reading, cold and warm were well distributed among both the migraine and tension type headache (TTH) patients. But significant difference was demonstrated for fatigue (p < 0.05), sleep deprivation (p < 0.05), sunlight (p < 0.01) and food (p < 0.05), which were common among migraineurs. In consideration of relieving factors of pain, different maneuvers were commonly tried by migraineurs and significant difference were observed for both analgesic drug and massage (p < 0.05), which relieved migraine headache. But maneuvers like sleep, rest and posture were used by both groups.

**Conclusion:**

The most frequent precipitating factors for headache appear to be identical for both migraine and TTH patients. Even though some factors like fatigue, sleep deprivation, sunlight and food significantly precipitate migraine and drug, massage are effective maneuver for relieving pain among migrianeurs.

## Background

Headache is the commonest neurological condition in terms of number of people affected [1]. A number of studies have reported that headache sufferers claim factors precipitating or triggering their headache and list is not a short one (stress, emotion, flickering light, noise, fatigue, food, season etc.) [2,3]. But studies devoted to specific triggers related to migraine and tension type headache (TTH) are rare. Henry et al. [4] evaluated these triggers among migraineurs. Rasmussen [5] used an unique structured headache interview method. Robbins [6] gathered information on triggers at first visit to headache clinic. Peatfield [7] studied the relation of food with headache. Only Larmande et al [8] evaluated the climate as a possible precipitating factor. Martin et al [9] grouped these precipitators into two major classes; the negative effect (stress, anxiety, depression) and the visual disturbance (flicker, glare, eyestrain).Attention to trigger factors plays a prominent role in the clinical management of headaches. Schulman and Silberstein [10] advise, for example, that “wherever possible, practitioners should help susceptible patients learn to avoid triggers”, and Skaer [11] states that “migraine prevention is best achieved by avoidance of known migraine triggers”. The logic of this advice is very clear and, of course, if individuals could avoid any factor that could trigger a headache then they would not get a headache. In the practice of headache management, precipitating factors are established for patients to avoid. They also provide insight into the mechanisms involved in the headaches, facilitating diagnosis. The question addressed in this study was as to see whether there are precipitating and relieving factors that separate the two most common headache conditions, ie, migraine and tension-type headache.

## Methods

This is an observational study carried out in weekly Headache outdoor clinic of Department of Neurology, DMCH. We reviewed the records of 250 migraine and same number of tension type headache (TTH) patients. All the patients were diagnosed clinically following the International Classification of Headache Disorder version-2 (ICHD-II) criteria by consultant neurologists at the clinic. The clinical diagnosis was considered as gold standard, considering the detail history of headache, examination findings and the vast clinical experience of the consultant neurologist in diagnosing headache disorder with the support of ICHD classification criteria. All the subtypes of migraine (migraine with aura, without aura, chronic migraine, probable migraine) were considered under the broad terminology of migraine headache. We included only those patients in whom headache could be classified convincingly either as migraine or TTH. Any patient of headache disorder with comorbid illness or more than one type of headache (combination of migraine and TTH, medication over use) were excluded from the study. We have used a predetermined check list of 11 established precipitating factors and 5 relieving factors for headache (Table 1). The study protocol was approved by the ethical committee of Dhaka Medical College hospital. Data were collected from hospital records by preformed questionnaire containing information on age, sex, social status, precipitating and relieving factors. Data was analyzed using SPSS-16 system.

**Table 1:** List of headache precipitating and relieving factors

**Precipitating factors:**	
1. Fatigue	
2. Stress	
3. Anxiety	
4. Cold	
5. Warm	
6. Sunlight	
7. Sleep deprivation/Insomnia	
8. Food	
9. Activity	
10. Journey	
11. Reading	
**Relieving factors:**	
1. Sleep	
2. Drug	
3. Rest	
4. Posture	
5. Massage	

## Result

In this study we divided all the 500 headache patients equally into two groups: migraine and TTH, following the international headache society criteria. All the precipitating and relieving factors we looked for are listed in (Table 1). The female patients predominated in this study (67% female and 33% male). Most of the patients were within 21–30 years age group (58.6%), followed by 18.2% patients within 11–20 years. Only 1.6% patients were below 10 years and 0.4% above 60 years of age (Table 2). About 58% of them belonged to middle class families and 40.6% from lower socioeconomic back ground (Table 2). The distribution of precipitating factors is listed in (Table 3). Most of the patients reported multiple precipitating and relieving factors. The common precipitating factors like stress (81, 96), anxiety (68, 51), activity (34, 24), journey (58, 46), reading (11, 14), cold (24, 31) and warmth (16, 24) were well distributed among both the migraine and TTH patients. But significant difference was demonstrated for fatigue (*p* < 0.05), sleep deprivation (*p* < 0.05), sunlight (*p* < 0.01) and food (*p* < 0.05). The relieving factors are enlisted in (Table 4). Significant difference were observed for both drug (152, 126) and massage (17, 6) (*p* < 0.05), which relieved migraine headache. But factors like sleep (146, 131), rest (25, 21) and posture (4, 1) were well distributed within both groups. Interestingly, the migraineurs tried each maneuver for headache remedy more frequently than the TTH patient except for the massage.

**Table 2:** Socio demographic profile of the patients (n = 500)

**Parameter**	**n**	**%**
**Age**		
<10yr	8	1.6
11-20yrs	91	18.2
21-30yrs	293	58.6
31-40yrs	69	13.8
41-50yrs	22	4.4
51-60yrs	15	3
>60yrs	2	0.4
**Sex**		
Male	165	33
Female	335	67
**Socio-economic status**		
Lower	203	40.6
Middle	90	58
Upper	7	1.4

**Table 3:** Comparison of common precipitating factors among migraine and TTH

**Precipitating factor**	**Migraine (%)**	**TTH (%)**	***p*value**
Sunlight	110 (44)	70 (28)	<0.01
Fatigue	34 (13)	18 (7)	<0.05
Sleep deprivation	52 (20)	35(14)	<0.05
Stress	81 (32)	96 (38)	>0.05
Anxiety	68 (27)	51 (20)	>0.05
Cold	24 (9)	31 (12)	>0.05
Warm	16 (6)	24 (9)	>0.05
Activity	34 (14)	24 (9)	>0.05
Journey	58 (23)	46 (18)	>0.05
Food	7 (2.8)	1(0.4)	<0.05
Reading	11 (4)	14 (5)	>0.05

**Table 4:** Comparison of common relieving factors among migraine and TTH

**Relieving factor**	**Migraine (%)**	**TTH (%)**	***p*value**
Drug	152 (61)	126 (50)	<0.05
Sleep	146 (58)	131 (52)	>0.05
Massage	17 (7)	6 (2.4)	<0.05
Rest	25 (10)	21(8.4)	>0.05
Posture	4 (1.6)	1 (0.4)	>0.05

## Discussion

Different precipitating factors for headache that we studied had been described by several authors previously [12,13]. In this study we have observed that several precipitating factors were identical among both migraineurs and TTH patients. Factors related to endogenous psychogenic mechanism like stress, anxiety were well distributed among both groups as precipitator. Rasmussen [5] also reported stress and mental tension as most frequent precipitants for both migraine and tension type of headache. In a study of 494 patients with migraine headache Robbins L [6] et al. observed similarly that stress was the most cited precipitating factor. Stress/ anxiety does so by central mechanism through direct activation of the ascending reticular pathway. Other factors like journey, physical activity, exposure to cold/warm, reading were also common in both group of patients and did not show any significant difference. Contrary to this finding, in a population based study in Croatia Zivadinov R [14] et al. showed that stress was associated with migraine whereas physical activity was related to TTH. Journey, change in weather and temperature were also associated among patient of migraine with aura in his study. The difference might account on the social and environmental variation among population. Similar to Spierings ELH et al. [15] we didn’t find any precipitating factor that was significantly reported frequently by TTH patients than the migraineurs. Instead fatigue, deprivation from sleep, sunlight and food were more frequently indicated significantly by migraineurs than the TTH patients (p <0.05 in all of the factors). Our finding is also supported by the report of Chabriat H et al. [16] who showed that fatigue, sleep difficulty and food or drinks more frequently precipitate headache among migraineurs than the nonmigraneurs. Deprivation of sleep results in fatigue which in turn, activates the sympathetic outflow to boost metabolic process for availability of energy. The sympathetic activation subsequently thought to precipitate headache.

Most of the people with headache disorders practice many nonpharmacological measures to get the relief of the pain. However, it is not known whether behavior during the attack is headache-type-specific or a general response to head pain. Martins and Prarreira [17,18] identified six maneuvers tried by the patients, most commonly the migraineurs to improve the headache during and attack. It is a common observation, by clinicians involved in the headache field that many patients use some instinctive manoeuvres, of their own accord that tend to alleviate their suffering. In our quest to identify the relieving factors we also observed that the rates of use of each maneuver were more frequent for the migraineurs, though the difference was not significant except for drug and massage. Other factors like sleep, rest and posture were well used by both groups. Both use of drug and massage was associated with relieve of pain in migraneurs. But this is quite contrary to the report of Bag B et al. [19] where massage relived pain among TTH patients. Unlike our observation, they also showed sleep, rest, change in posture were also significantly relieved pain with migraine patients.

We had some limitations in this study. Firstly, we could not match the age and sex among the two groups of patients due to wide variation of age and sex specific prevalence of headache and the retrospective nature of the study. Secondly, the chance of recall bias was minimized by the check list of precipitating and relieving factors in hospital records. Finally, the chance of observer biasness in diagnosis of headache disorder was also minimized as they followed the international headache society criteria (ICHD-II 2004) at the specialized headache clinic.

## Conclusion

Most of the precipitating and relieving factors are common and similar in both migraine and TTH patients. Though some of them (fatigue, sleep deprivation, sunlight and food) are indicated significantly among migraineurs, there is no factor associated significantly with TTH. This difference is also true for relieving factors (drug and massage relived migraine headache). The careful monitoring of the precipitating factors of headache could be an important step in treatment, because their avoidance may lessen the frequency and severity of attacks. Furthermore, they may also provide a clue to the aetio-pathogenesis of headache. So the search for these precipitating and relieving factors should be continued in future.

## Competing interest

The authors declare that they have no competing interests.

## Author’s contributions

The first three authors were involved in patient consultation, data collection and writing the article. The corresponding author was involved in data entry, data analysis and writing the article. The rest of the authors were involved in patient consultation and data collection step of the research. All authors read and approved the final manuscript.

## Funding

The study was not funded by any donor agency.

## Pre-publication history

The pre-publication history for this paper can be accessed here:

http://www.biomedcentral.com/1471-2377/12/82/prepub
